# Management of an endo perio lesion in a maxillary canine using platelet-rich plasma concentrate and an alloplastic bone substitute

**DOI:** 10.4103/0972-124X.55839

**Published:** 2009

**Authors:** Sangeeta Singh

**Affiliations:** *Graded Specialist (Periodontology), 320 Field Hospital, C/O 99 APO, Pin - 903320, India*

**Keywords:** Endoperio lesion, platelet-rich plasma, infrabony defect

## Abstract

To evaluate the efficacy of platelet-rich plasma concentrate in the management of a cirumferential, infrabony defect associated with an endoperio lesion in a maxillary canine. A 45 year-old male patient with an endoperio lesion in the left maxillary canine was initially treated with endodontic therapy. Following the endodontic treatment, the circumferential, infrabony defect was treated using platelet-rich plasma and an alloplastic bone substitute. At the end of three months, there was a gain in the clinical attachment level and reduction in probing depth. Radiographic evidence showed that there was significant bony fill. The results were maintained at the time of recall nine months later.

## INTRODUCTION

Establishing an accurate diagnosis is the first step to success in endodontic therapy. In many cases, the diagnosis is easy to establish, but there are certain cases where the situation becomes more complex, especially when it co-exists with periodontal disease. It is well known that clinical endodontic diagnosis is based on the patient's history, clinical evaluation and the radiographic appearance of the lesion. Diagnosis of necrotic pulps may be difficult to establish because symptoms and signs may be inconclusive and poorly localized.

The relationship between periodontal and pulpal disease was first described by Simring and Goldberg in 1964.[1] Since then, the term, ‘perioendo lesion’ has been used to describe lesions due to inflammatory products found in varying degrees in both the periodontium and the pulpal tissues. The pulp and periodontium have embryonic, anatomic, and functional interrelationships. They are ectomesenchymal in origin and proliferate to form the dental papilla and follicle, which are the precursors of the pulp and periodontium respectively. These are separated by the formation and development of the tooth bud from the overlying ectoderm into the enamel and dentine. Embryonic development gives rise to anatomical connections which remain throughout the life of the tooth[2]

The apical foramen decreases in size as the proliferation of the Sheath of Hertwig continues. It remains patent and serves as the communication on which the pulpal tissues rely for nutrition and nervous innervation. As the root develops, ectomesenchymal channels get incorporated, either due to dentine formation around existing blood vessels, or breaks in the continuity of the Sheath of Hertwig, to become accessory or lateral canals. The majority of accessory canals are found in the apical part of the root and lateral canals in the molar furcation regions. Tubular communication between the pulp and periodontium may occur when dentinal tubules become exposed to the periodontium due to the absence of overlying cementum. These are the pathways that may provide the means by which pathological agents pass between the pulp and periodontium, thereby creating the perioendo lesion.[3,4]

Intrabony defects associated with deep periodontal pockets, are ecological niches for periodontal pathogens, and if left untreated, they complicate the outcome of any therapy performed on the tooth. In most cases of endoperio lesions, clinical symptoms disappear following successful endodontic therapy. However, it becomes essential to correct the periodontal defect simultaneously in these cases to prevent recurrence, and to improve the functional status of the tooth.[5]

## CASE REPORT

**Figure 1:**
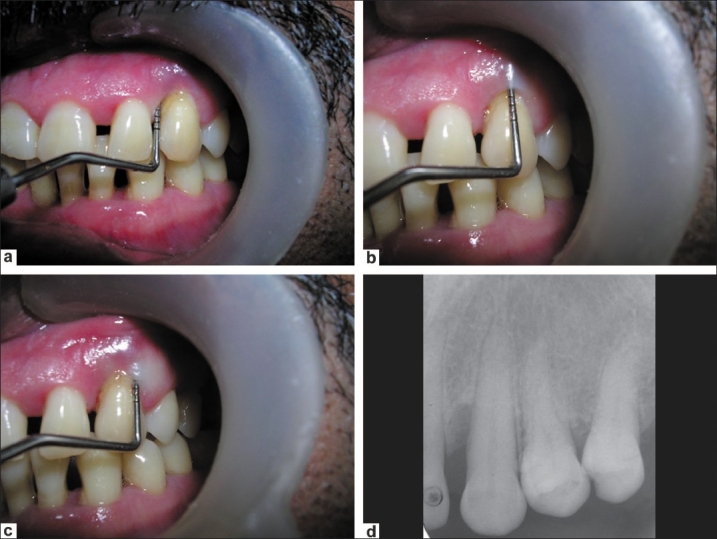
a) Periodontal probing depth was 8 mm mesially; b) Probing depth of 6 mm labially; c) Probing depth of 7.5 mm distally; d) A periapical radiograph showed a widening of the periodontal ligament space in the periapical area with an infrabony defect on the mesial aspect

A 45 year-old male patient reported with the chief complaint of pain, swelling, and pus discharge from the maxillary left canine of one month's duration. The tooth was previously sensitive to hot and cold, and had recently developed spontaneous pain and pus discharge. Periodontal probing depths were 8 mm mesially, 6 mm labially, and 7.5 mm distally [Figure 1a–c]. A periapical radiograph showed a widening of the periodontal ligament space in the periapical area with an infrabony defect on the mesial aspect of the tooth [Figure 1d]. An endoperio lesion associated with maxillary left canine was diagnosed.

**Figure 2:**
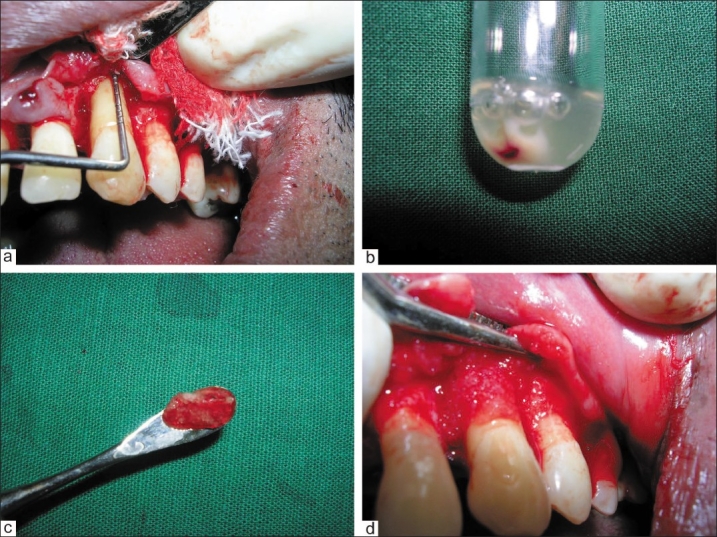
a) The site was surgically opened up for debridement and a circumferential defect was evident around the tooth; b) The platelet-rich concentrate obtained after centrifugation; c) The platelet-rich concentrate was mixed with an alloplastic bone graft substitute and the mix obtained had a gel-like consistency; d) This gel was placed to cover the exposed root and fill the defect

An access cavity was prepared and the root canal system was cleaned and shaped in the first session with abundant 5.25% sodium hypochlorite irrigation. The canal was dressed temporarily with calcium hydroxide and the access cavity sealed with IRM cement. The patient was asked to stop all analgesic drugs and was given an appointment in seven days to continue the root canal treatment. He was also asked to contact the dental centre if there were any complaints. He was completely comfortable without any need for analgesia. The patient returned after a week and the absence of pain or signs of inflammation indicated that the final filling could be placed. This was completed with gutta-percha and a root canal sealer. A one-month recall revealed a stable situation and disappearance of pain, however, the pockets persisted around the tooth. It was decided to correct the defect after one month after the endodontic therapy, using autologous platelet concentrate mixed with an alloplastic bone graft substitute. The site was surgically opened up for debridement and a circumferential defect was evident around the tooth [Figure 2a]. The platelet-rich concentrate was mixed with an alloplastic bone graft substitute to obtain a gel-like consistency [Figure 2b and c]. This gel was placed to cover the exposed root and fill the defect [Figure 2d].

**Figure 3:**
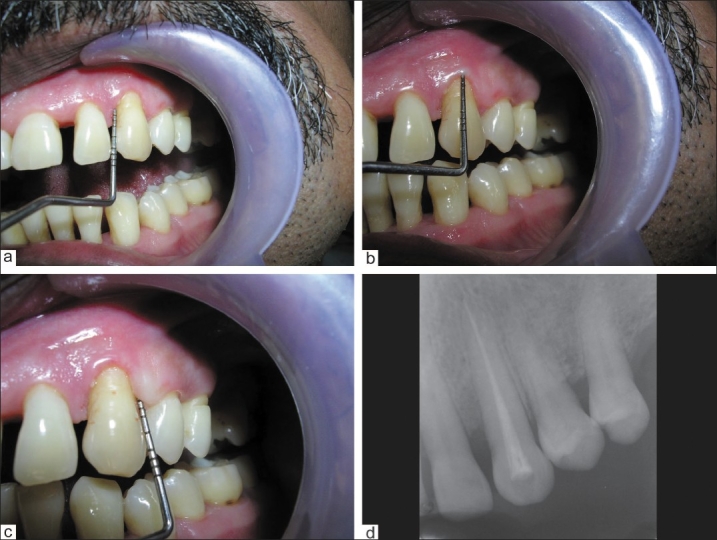
a) The periodontal pockets had reduced from 8 mm to 0.5 mm mesially; b) The periodontal pockets had reduced from 6 mm to 1 mm labially; c) The periodontal pockets had reduced from 7.5 mm to 1 mm distally; d) Radiographically, a significant bony fill was evident after nine months

The clinical appearance of the tooth had improved considerably at the time of evaluation three and six months following treatment. The periodontal pockets had reduced from 8 mm to 0.5 mm mesially, from 6 mm to 1 mm labially, and from 7.5 mm to 1 mm distally [Figure 3a–c]. Radiographic evidence showed a significant bony fill [Figure 3d]. The results were stable and maintained at the end of nine-months' follow-up.

## DISCUSSION

Endoperio lesions are common conditions that are often difficult to diagnose and persistent if not treated completely. However, if the patient's history is taken carefully and thorough evaluation of all possible routes of infection is carried out, these lesions can be completely eliminated to give excellent results. A correct diagnosis helps in formulating a correct treatment plan and in most of the cases, a properly done endodontic treatment is sufficient to eliminate the infection. However, wherever a secondary periodontal involvement exists, it requires specific therapy to achieve success. Most of these lesions have a vertical osseous defect and regenerative therapy gives excellent results after open flap debridement.

Periodontics has come a long way from the era of Schallhorn who described the use of an iliac crest autograft to treat periodontal defects. Today, there are various options available to treat periodontal defects. However, autologous platelet concentrate is a very novel technique which has proved to be successful in the management of infrabony defects.

The effects of platelet concentrate have been examined *in vitro* and *in vivo*. In 1995, Slater *et al*.[6] added human platelet concentrate to fetal calf serum medium containing human fetal osteoblasts. They demonstrated stimulated proliferation and maintained the differentiated function of the cells in tissue culture. Marx *et al*. added autologous platelet-rich plasma to cancellous cellular marrow bone grafts to repair mandibular discontinuity defects.[7]

Wound healing commences with hemostasis, which includes the formation of a fibrin clot, platelet adhesion, and aggregation. In the process of aggregation, alpha granules of platelets release many mediators, including platelet-derived growth factor (PDGF) and transforming growth factor (TGF)-α and -β. These growth factors promote fibroblast chemotaxis (PDGF and TGF-β), proliferation (PDGF), contraction (PDGF), extracellular matrix deposition (TGF-β), and reepithelialization (TGF-α) in the healing wound.[1] Periodontal ligament fibroblasts, cementoblasts, and osteoblasts are affected similarly by these growth factors.[8]

This case of a patient with an endoperio lesion who was treated endodontically and followed by regenerative therapy to treat the circumferential defect, emphasizes the need for careful evaluation of complicated cases where conventional therapy fails due to incomplete elimination.

## CONCLUSION

In this case, successful treatment can be attributed to a correct diagnosis, successful endodontic therapy, and bone fill achieved due to the use of autologous platelet concentrate.
